# Phthisis Pulmonalis as a Sequel to Suppressed Menstruation

**Published:** 1867

**Authors:** 


*Phthisis Pulmonalis as a Sequel to Suppressed Menstruation.*

It is not unusual to hear it remarked, that the suppression of the catamenial discharge is the cause of phthisis pulmonalis, by which it is frequently accompanied. Nor is it strange, when we come to consider, that several eminent authors give countenance and support to this same statement. But the question arises, is this a fact, or is it a mere assertion, sanctioned by time, and reverenced, like owls, on account of its antiquity? Let prejudice and preconceived notions be laid aside, and let us examine, for a moment, whether the one, as an effect, has any relation to the other, as a cause. It is now generally admitted that the development of phthisis is preceded by, and depends upon, a constitutional vice, a peculiar diathesis, a cachectic state of the system, either inherited or acquired. That it is often inherited from parents and transmitted through several generations, is not denied, but some have doubted as to the possibility of its being acquired. Modern researches, however, have settled the question beyond a doubt, and it is now generally believed that the tubercular diathesis may be produced by those influences which tend to impair nutrition and assimilation, to deprave the blood, by wasting the red globules and changing, to some extent, its normal constitution.

Now, the question arises, will the suppression of the menses bring about this diathesis, or will it even develope the disease, when the diathesis exists? If we examine the discharge, we will find it to be composed of blood, mingled with a small amount of the ordinary secretion of the parts. That it consists of nothing more, is evident from several considerations:—1st. When chemically examined, nothing of an excrementitious or poisonous nature is detected in its composition. 2d. The organ from whence it flows is not, like the skin or kidneys, designed to eliminate matter from the system which, if retained, would be productive of injury. 3d. The function, which is only temporary, after having ceased entirely, is not followed by the disease under consideration. Now, if the menstrual discharge contains nothing injurious to the system, no harm can possibly ensue from its mere retention in the system, neither can the mere retention be productive of any injury beyond slight constitutional disturbance. It is wholly and entirely inadequate to produce those fearful results which we designate as phthisis pulmonalis.

But it may be affirmed, that the suppressing the menstrual discharge does produce many very serious phenomena, and that this is a matter of daily observation, and cannot be denied. It is no doubt true, that the suppression is followed or accompanied by these various phenomena, but they are not due to the fact that the function is not performed, but to the cause which produced the suppression. Suppose, for example, that the catamenia fail to appear, od account of cold, is it not possible that this very cold may check excretion of the skin, and the kidneys failing to take upon themselves vicarious duty, matter may be retained in the system which should be eliminated, and all thc various phenomena be produced which are usually attributed to the failure of the catamenia?

But it may be asked, how do we account for the fact that the suppression of the catamenia, for some length of time, is often followed by phthisis? The reason is obvious. The same causes which tend to produce suppression of the menstrual function, tend also to develope the tubercular diathesis, or to call it into action when it already exists. Take, again, the action of cold, which is a very common cause of suppression, is it not also one of the most exciting causes of phthisis? Again, the menstrual function may cease to be carried on from mere debility, produced, perhaps, by previous disease, or by a low state of living, food poor in quality and deficient in quantity, foul atmosphere, damp, crowded, and ill-ventilated apartments, such a state of life as is often witnesed among the extreme poor in crowded cities. Are not these very circumstances most favorable to the development of the tubercular diathesis? And are not both the suppression and the consumption due to the same causes?

It is surely reasonable to infer that the tubercular disease is not owing to the suppression of the menstrual function, but to the debility produced by these various causes which have so depraved the blood and impaired the vital energy, as to cripple the resources of nature and render her unable to resist the stealthy and insidious approaches of this most formidable disease.

